# Retraction Note: FAM83H-AS1/miR-485-5p/MEF2D axis facilitates proliferation, migration and invasion of hepatocellular carcinoma cells

**DOI:** 10.1186/s12885-022-09835-3

**Published:** 2022-07-05

**Authors:** Wenpeng Zhao, Jiang Guo, Honglu Li, Liang Cai, Youjia Duan, Xiaopu Hou, Zhenying Diao, Xihong Shao, Hongliu Du, Changqing Li

**Affiliations:** Department of Oncology Interventional Radiology, Beijing Ditan Hospital, Capital Medical University, No.8 Jingshundong Road, Beijing, 100015 China

**Retraction Note: BMC Cancer 21, 1310 (2021)**

**https://doi.org/10.1186/s12885-021-08923-0**

The Editor has retracted this article at the corresponding author's request. After publication, the authors became aware that their MHCC-97H and HCCLM3 cell lines were contaminated with HepG2, which affected the results of the FAM83H-AS1 overexpression experiment. The authors therefore no longer have confidence in the conclusions of this article.

All authors agree to this retraction.

